# Transcriptome and metabolome analysis of plant sulfate starvation and resupply provides novel information on transcriptional regulation of metabolism associated with sulfur, nitrogen and phosphorus nutritional responses in Arabidopsis

**DOI:** 10.3389/fpls.2014.00805

**Published:** 2015-01-28

**Authors:** Monika Bielecka, Mutsumi Watanabe, Rosa Morcuende, Wolf-Rüdiger Scheible, Malcolm J. Hawkesford, Holger Hesse, Rainer Hoefgen

**Affiliations:** ^1^Department of Pharmaceutical Biotechnology, Faculty of Pharmacy, Wroclaw Medical UniversityWroclaw, Poland; ^2^Max-Planck Institute of Molecular Plant PhysiologyPotsdam-Golm, Germany; ^3^Institute of Natural Resources and Agrobiology of Salamanca, Consejo Superior de Investigaciones CientíficasSalamanca, Spain; ^4^Plant Biology Division, The Samuel Roberts Noble FoundationArdmore, OK, USA; ^5^Rothamsted Research, Plant Biology and Crop Science DepartmentHarpenden, UK

**Keywords:** sulfur, sulfate starvation, nitrate, phosphate, transcription factor, microarray, metabolomics, transcriptomics

## Abstract

Sulfur is an essential macronutrient for plant growth and development. Reaching a thorough understanding of the molecular basis for changes in plant metabolism depending on the sulfur-nutritional status at the systems level will advance our basic knowledge and help target future crop improvement. Although the transcriptional responses induced by sulfate starvation have been studied in the past, knowledge of the regulation of sulfur metabolism is still fragmentary. This work focuses on the discovery of candidates for regulatory genes such as transcription factors (TFs) using ‘omics technologies. For this purpose a short term sulfate-starvation/re-supply approach was used. ATH1 microarray studies and metabolite determinations yielded 21 TFs which responded more than 2-fold at the transcriptional level to sulfate starvation. Categorization by response behaviors under sulfate-starvation/re-supply and other nutrient starvations such as nitrate and phosphate allowed determination of whether the TF genes are specific for or common between distinct mineral nutrient depletions. Extending this co-behavior analysis to the whole transcriptome data set enabled prediction of putative downstream genes. Additionally, combinations of transcriptome and metabolome data allowed identification of relationships between TFs and downstream responses, namely, expression changes in biosynthetic genes and subsequent metabolic responses. Effect chains on glucosinolate and polyamine biosynthesis are discussed in detail. The knowledge gained from this study provides a blueprint for an integrated analysis of transcriptomics and metabolomics and application for the identification of uncharacterized genes.

## Introduction

Plants have a constitutive demand for sulfur to synthesize sulfur amino acids, numerous essential metabolites and secondary metabolites for growth and development. Sulfur deficiency in crops has become an increasing problem in many countries, notably in Western Europe because it causes growth retardation, earlier flowering and chlorosis, which result in depression of yield, nutritional quality and taste of crops (Schnug, 1991; Zhao et al., 1993; McGrath et al., 1996; Marincs et al., 2006). Studies of sulfur acquisition and metabolism in plants have become a major concern for research and crop improvement.

During the past decades, remarkable progress has been made in the basic understanding of regulatory mechanisms, genes and proteins involved in sulfur assimilation. Studies with different model organisms, such as *Escherichia coli* (Phillips et al., 1989; Augustus et al., 2006; Lamonte and Hughes, 2006; Marincs et al., 2006), *Saccharomyces cerevisiae* (Cherest et al., 1969, 1985; Thomas et al., 1990, 1992a, b; Kuras et al., 1996, 1997; Thomas and Surdinkerjan, 1997), *Neurospora crassa* (Fu et al., 1989; Paietta, 1990, 1992, 1995; Marzluf, 1997; Sizemore and Paietta, 2002), *Aspergillus nidulans* (Mountain et al., 1993; Paszewski, 1999; Piotrowska et al., 2000; Natorff et al., 2003) and *Chlamydomonas reinhardtii* (Dehostos et al., 1988; Davies et al., 1994, 1996; Yildiz et al., 1994, 1996; Ravina et al., 1999, 2002; Pollock et al., 2005), contributed to our understanding of regulatory processes for sulfur assimilation. Nevertheless, the transcriptional regulation of sulfate assimilation in plants remains incomplete, mostly because orthologous genes of the corresponding regulatory factors have not been found. Thus, the complex signaling pathways of plants which regulate sulfur metabolism are not yet fully understood though some progress has been achieved recently.

System-wide descriptions using ‘omic studies such as transcriptomics, proteomics, and metabolomics provide tools for the identification of potential target genes and metabolic processes underlying the physiological response of a plant to varied nutrient availability (Hirai and Saito, 2004). Reports published to date describing the transcript profiles of sulfate starved Arabidopsis plants have been limited to DNA macro- or microarrays that represented around 8000 (Maruyama-Nakashita et al., 2003; Nikiforova et al., 2003) or 9000 genes (Hirai et al., 2003) due to the early stage of development of these technologies. Detailed comparison of data from array experiments and metabolic profiles were performed (Hirai and Saito, 2004; Nikiforova et al., 2004, 2005a). By combining these results, response networks during sulfate starvation were proposed in addition to the expected direct effects on sulfate uptake and assimilation pathways. However, no transcription factor (TF) was identified that was common in more than two studies (Hirai and Saito, 2004). This is assumed to be due to the fact that (i) each array used in these studies contained only approximately one-third of all Arabidopsis genes and that (ii) the sensitivity of the arrays was not high enough as the expression levels of TFs are expected to be low, and (iii) the experimental setups were quite diverse, probably affecting the final physiological responses depending on the severity of sulfate shortage, the plant growth stage, and the period of sulfate starvation. As a result, the most downstream genes in the signal transduction pathway were different from experiment to experiment. It should also be taken into consideration that commonly the transcript abundance for specific regulators does not change under conditions in which the regulator functions to alter downstream gene expression, making it impossible to identify these factors in a differential screening (Davies et al., 1999). Indeed, glucose-deprived yeast cells and sulfur-deprived Chlamydomonas do not exhibit altered transcript levels of the regulators SNF1 (Celenza and Carlson, 1984) and Sac3 (Davies et al., 1999), respectively. Also SLIM1, a TF in Arabidopsis, which controls both the activation of sulfate acquisition and degradation of glucosinolates under sulfate starved conditions, was reported to be not regulated itself by these conditions at the transcript level (Maruyama-Nakashita et al., 2006). SLIM1 was identified in a genetic approach, in which a series of Arabidopsis mutants with a sulfur limitation-responsive promoter-GFP reporter system were screened for attenuated sulfur limitation phenotypes.

In addition to SLIM1, R2R3-MYB TFs were identified as involved in the regulation of sulfur metabolism. MYB28, MYB76, and MYB29 are specifically involved in the control of synthesis of aliphatic methionine derived glucosinolates (Hirai et al., 2007; Sonderby et al., 2007; Gigolashvili et al., 2007a, 2008) while MYB51, MYB122, and MYB34 regulate synthesis of indolic tryptophan derived glucosinolates (Celenza et al., 2005; Gigolashvili et al., 2007b; Malitsky et al., 2008). It has been reported that these MYBs activate the sulfate reduction pathway, which is required for glucosinolate production (Yatusevich et al., 2010).

The incomplete differential transcriptomics due to previous technical limitations demands improved investigations using complete gene chips to provide more consistent information and interpretation than currently available. In order to identify genes with altered expression levels under sulfate limiting conditions, in addition to simple sulfate starvation, we applied two time points of re-supply in order to identify relaxation of the gene transcript responses, to differentiate between specific and unspecific or pleiotropic effects. The main focus of these experiments was placed on describing the system at the transcriptome and metabolome levels and with special emphasis on transcription factors, a major gap in our understanding of sulfur metabolism.

## Results

### Experimental setup and physiological features of arabidopsis seedlings grown in liquid cultures

Arabidopsis seedlings were grown in 30 mL of sterile liquid full nutrition (FN) medium (3 mM sulfate) or 150 μ M sulfate medium. The FN medium provided sufficient sulfate to the seedlings throughout the growing phase and was used as a control. The 150 μ M sulfate medium allowed seedlings to germinate and grow. After 7 days of cultivation, the sulfate in that medium was consumed by the plants and decreased to undetectable amounts. This forced plants to mobilize sulfate resources stored in their vacuoles. Transferring pre-grown 7-days old seedlings to a sulfate depleted medium (0 μ M sulfate) assured immediate and continued sulfate starvation during the next 2 days of plant cultivation. Nevertheless, the sulfate deprived seedlings did not exhibit typical phenology of sulfur limited plants, which are known to be: reduced chlorophyll which might be concomitant with senescence processes (Watanabe et al., 2010, 2013), accumulation of anthocyanins in the leaves, and effects on roots, especially lateral root growth (Nikiforova et al., 2003; Hubberten et al., 2012a). On day 9, all seedlings in both FN medium and sulfate depleted medium had developed cotyledons and first leaves which remained green. On day 9 subsets of the sulfate depleted cultures were supplied with sulfate (500 μ M) and samples taken 30 min and 3 h after re-supply. Two technical repetitions of each sample were grown and harvested to minimize the influence of flask handling. Eventually, four different samples corresponding to four time points [full nutrition (FN), plants starved for 48 h (-S), plants re-supplied with sulfate for 30 min (30' S) and plants re-supplied with sulfate for 3 h (3 h S)] were subjected to further analysis.

### Alteration in sulfur related metabolites and primary metabolites under sulfate-starved and re-supplied conditions

**Figure 1:**
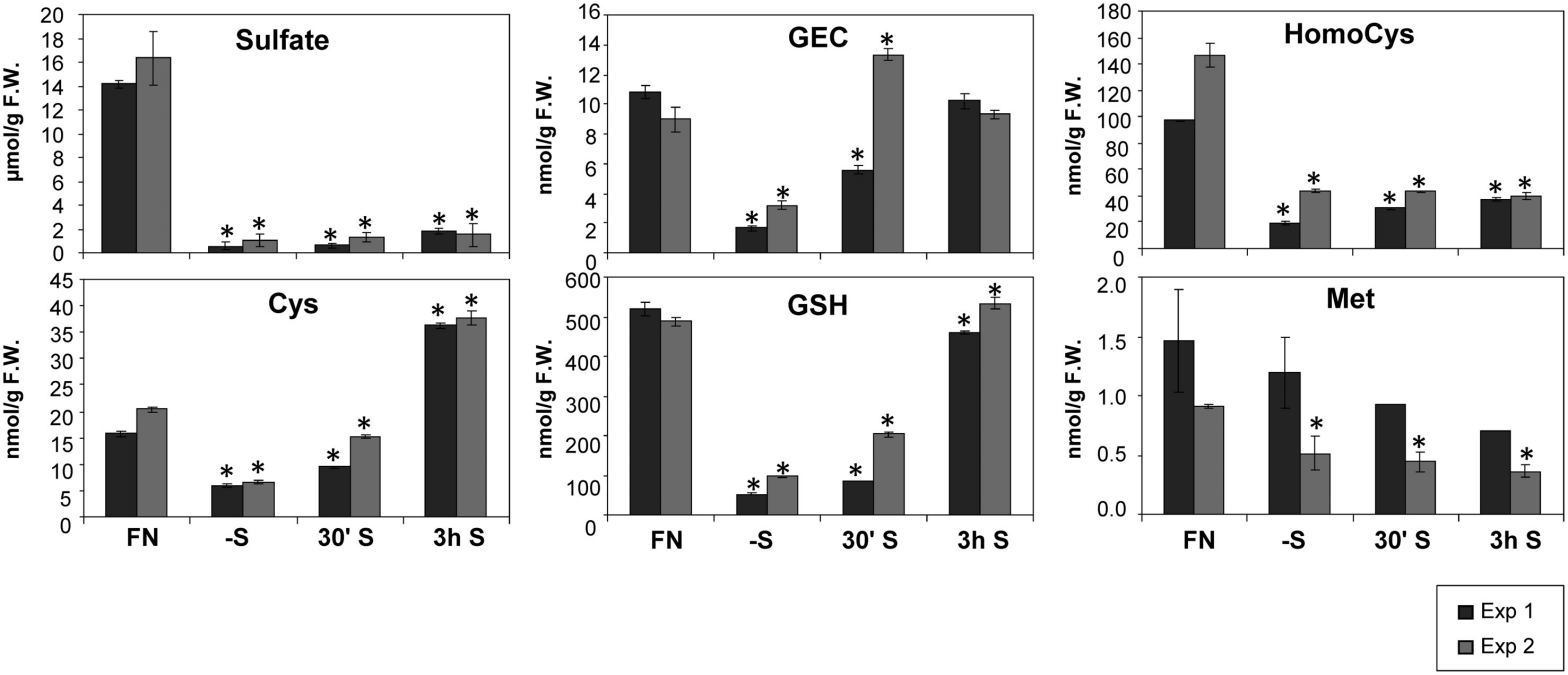
**Levels of sulfur related metabolites in Arabidopsis seedlings grown in liquid cultures under different sulfate regimes**. Values ± SD characterize the average of three independent measurements. Asterisks indicate values that are significantly different (*P* < 0.05) in comparison to the respective FN controls. (FN, full nutrition; S, re-supplied sulfate).

The tissue content of sulfate was determined as an indicator for the status of sulfate starvation of the plant. As expected, strong decreases of sulfate content after 2 days of sulfate starvation were observed (Figure 1). Starved seedlings contained only 4–6% of the sulfate level measured in control plants grown on FN medium. Despite re-supply of sulfate, only a slight 1.5-fold increase of sulfate was detected 3 h after re-supply in experiment 1, compared to continually starved plants. The content of organic sulfur compounds such as cysteine (Cys) and glutathione (GSH) were reduced upon sulfate starvation (Figure 1). In contrast to the slight changes of inorganic sulfate upon resupply, the levels of Cys and GSH were significantly increased after re-supply of sulfate (Figure 1). Interestingly, the GSH precursor gamma-glutamylcysteine (GEC) abundance peaks even before the accumulation of GSH, showing a delay in the conversion of GEC to GSH in experiment 2.

Despite the fact that Cys serves as a precursor of methionine through a transsulfuration reaction (Hesse and Hoefgen, 2003; Hesse et al., 2004a), methionine levels were not drastically affected by the limited input of sulfate in experiment 1, and by re-supply in both experiments 1 and 2. This is in accordance with previous data (Nikiforova et al., 2005b). Homocysteine as a direct precursor of methionine is a product of *de novo* synthesis from Cys in the plastids, and from *S*-adenosylmethionine (SAM) recycling as part of the SAM methylation cycle in the cytosol. SAM was found to be reduced strongly under sulfate starvation, while the cellular methionine levels remained constantly low in Arabidopsis (Nikiforova et al., 2005b). Homocysteine was significantly decreased under sulfate starvation indicating an effective conversion to methionine, which in turn feeds into SAM biosynthesis (Figure 1). The strong accumulation of the polyamine precursor putrescine (Table 1) indicates an insufficient availability of SAM under sulfate starvation conditions (Hanfrey et al., 2001). Moreover, sulfate starvation resulted in global changes in primary metabolites including perturbation of amino acid metabolism (Table 1), consistent with several reports published previously (Hirai et al., 2004; Nikiforova et al., 2005b). In contrast to the significant changes induced by sulfate starvation, re-supply of sulfate did not affect many of the changes in primary metabolites strongly after 30 min and 3 h (Table 1). In summary, metabolome data indicate that genes induced by sulfate starvation need more than 3 h to achieve a normal, un induced state; this needs to be taken into consideration when analyzing the data presented here, as the system did not yet reach homeostasis.

**Table 1:**
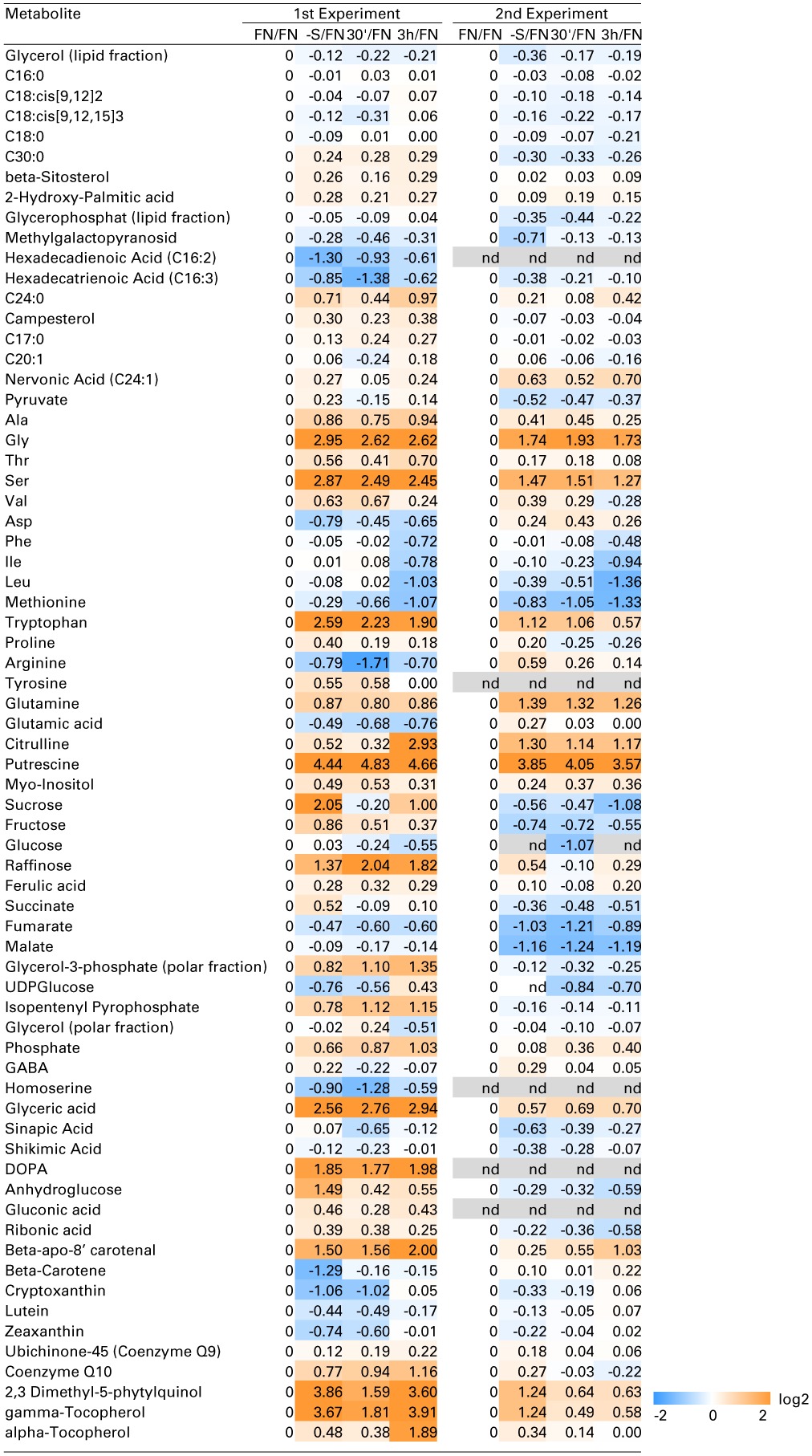
**Metabolic profile of sulfate deficiency in liquid culture experiments**.

*In most cases values represent the averages out of three independent measurements. FN, full nutrition; S, sulfate in medium; nd, not determined. Values and colors are in log2 scale*.

### Alteration in gene expression levels under sulfate-starved and re-supplied conditions

**Figure 2:**
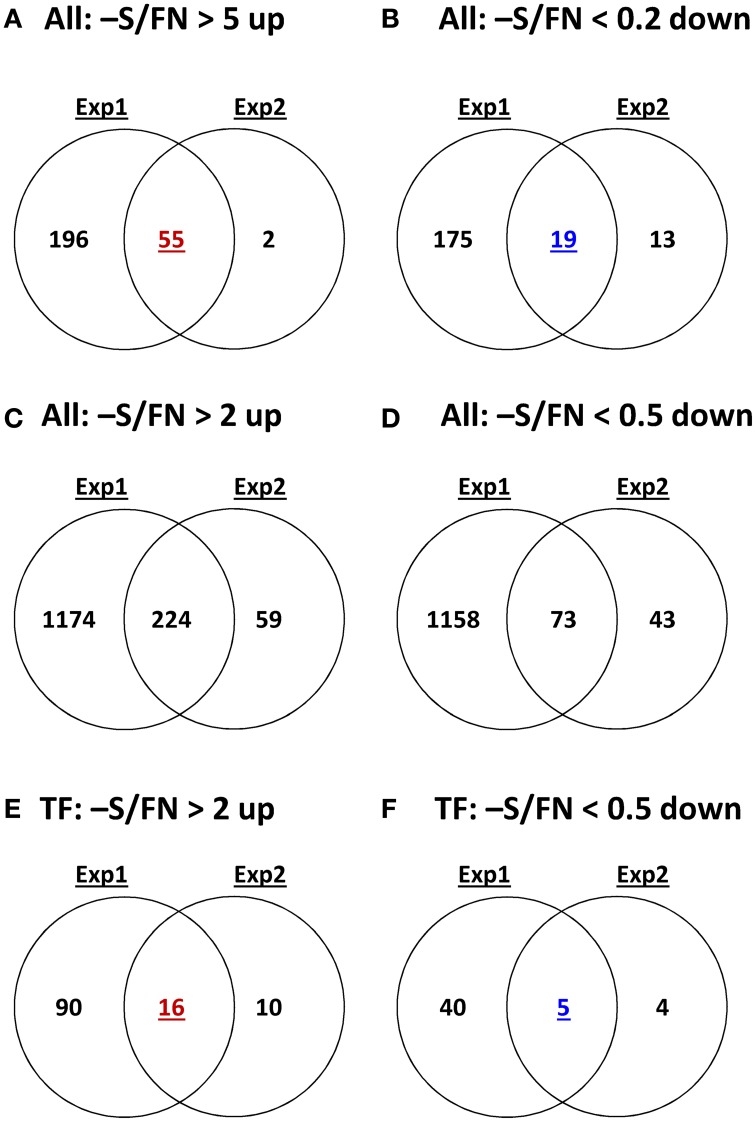
**Comparative analysis of the gene expression patterns under sulfate deficiency stress in two independent experimental replicates**. Venn diagrams show the numbers of genes more than 5-fold or 2-fold significantly up-regulated or down-regulated genes in all genes or TF genes with P and M calls on -S or FN condition. P, present value; M, marginal value.

**Figure 3:**
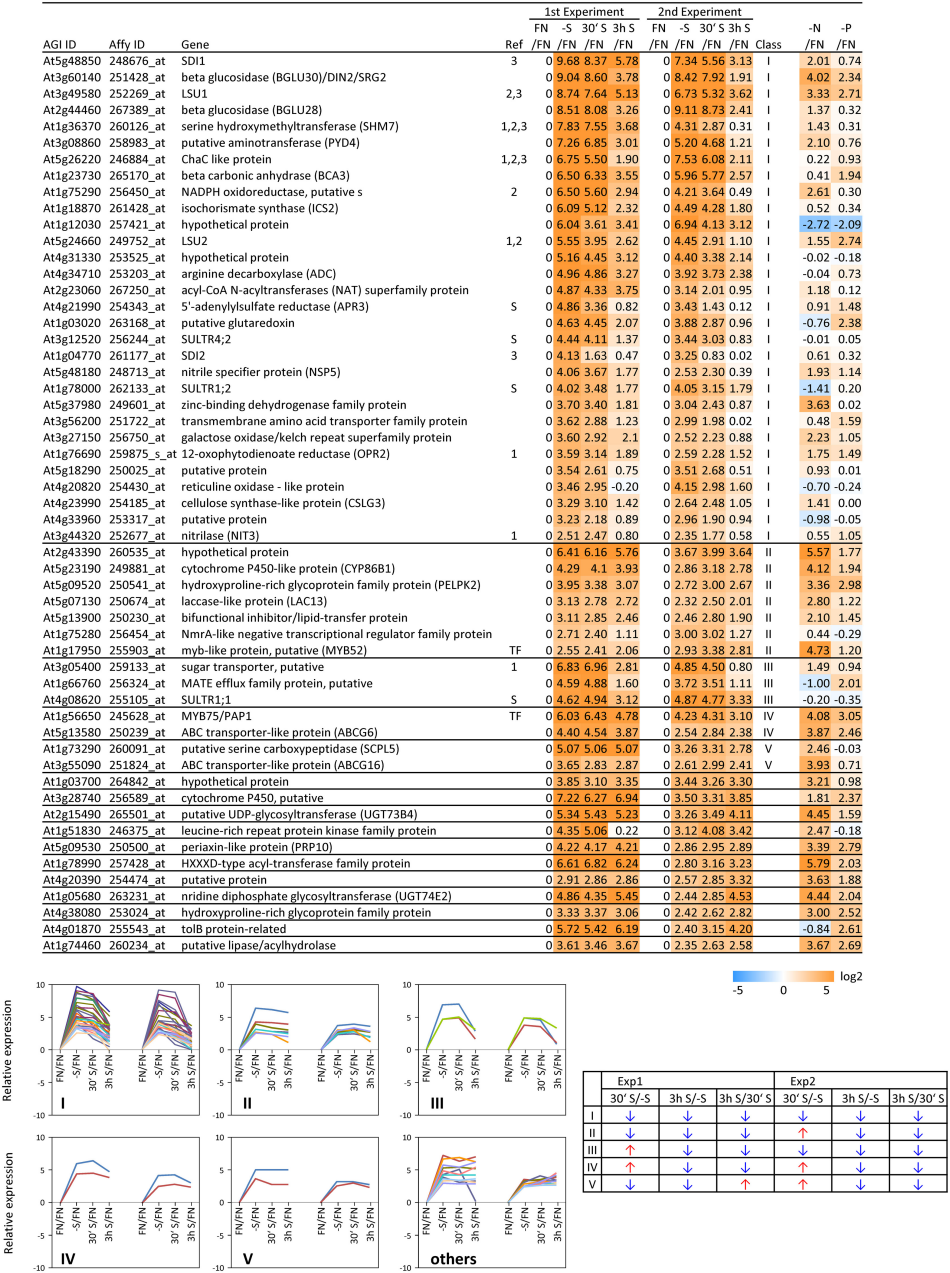
**Relative expression level of up-regulated genes under sulfate starvation**. Genes were up-regulated more than 5 times (55 genes) under sulfate deficient conditions in both experimental replicates. The up-regulated genes were classified into 6 clusters (class I–V, and others) according to responsiveness to sulfate resupply as indicated by fold changes (>1, upwards arrow; <1, downwards arrow) in transcript accumulations, 30' (30 min) S/-S, 3 h S/-S and 3 h S/30' S (Supplemental Table SIII). Genes already published as S-responding (Hirai and Saito, 2004) are marked with (1). Genes, in which promoters region (−3-kb upstream sequence) the SURE core sequence was found (Maruyama-Nakashita et al., 2005), are marked with (2). OAS responsive genes (Hubberten et al., 2012a, b), are marked with (3). Genes which are involved in sulfate uptake and reduction are marked in (S). Fold changes relative to the nitrate- and phosphate-sufficient control are shown. -N; data are from Scheible et al. (2004). -P; Morcuende et al. (2007). Values and colors are in log2 scale.

**Figure 4:**
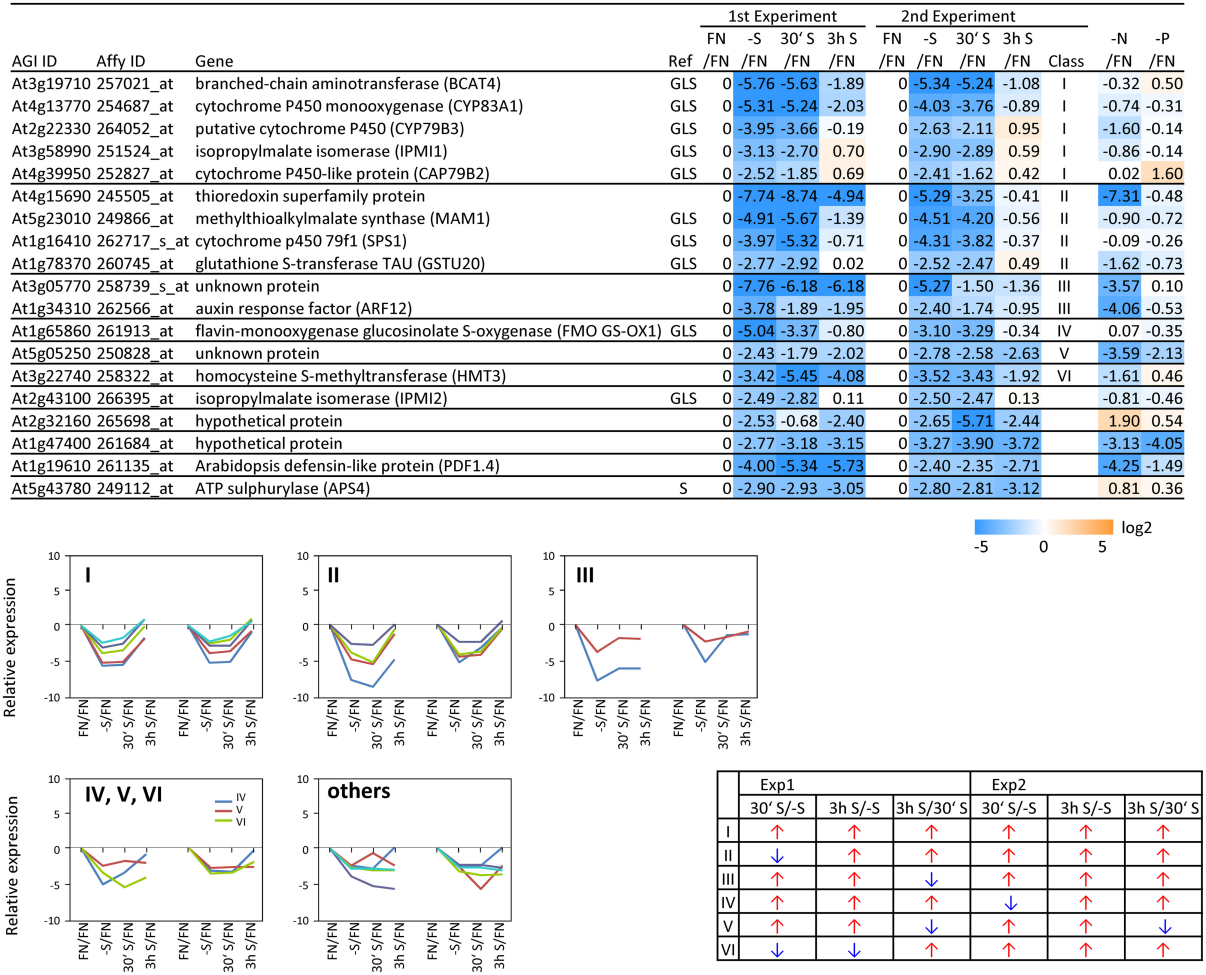
**Relative expression level of down-regulated genes under sulfate starvation**. Genes were down-regulated more than 5 times (19 genes) under sulfate deficient conditions in both experimental replicates. The down-regulated genes were classified into 7 clusters (class I–VI, and others) according to responsiveness to sulfate resupply as indicated by fold changes (>1, upwards arrow; <1, downwards arrow) in transcript accumulations, 30' (30 min) S/-S, 3 h S/-S and 3 h S/30' S (Supplemental Table SIII). Genes which are involved in sulfate reduction pathway and glucosinolate (GLS) biosynthetic pathway are marked with S and GLS, respectively. Fold changes relative to the nitrate- and phosphate-sufficient control are shown. -N; data are from Scheible et al. (2004). -P; Morcuende et al. (2007). Values and colors are in log2 scale.

**Figure 5:**
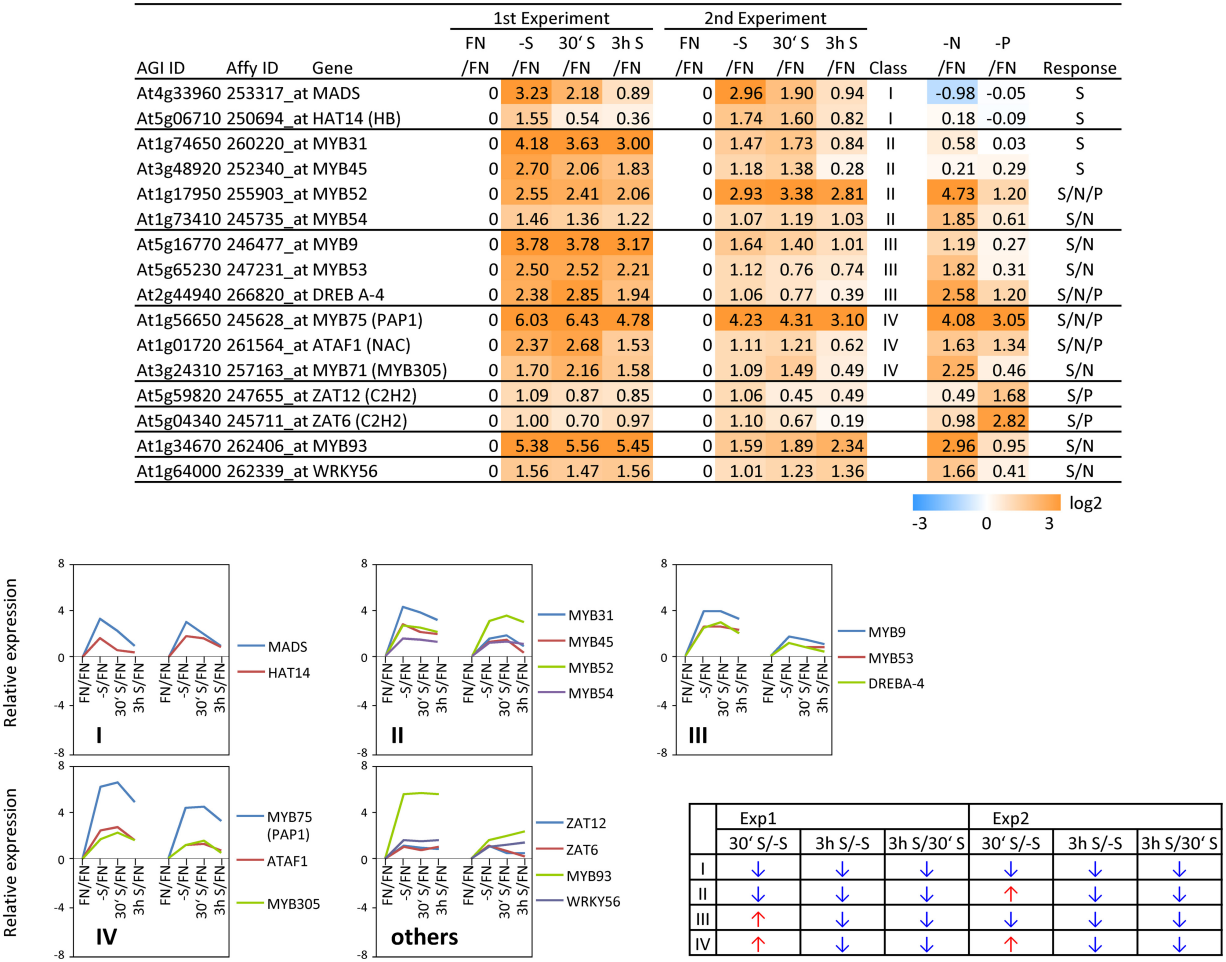
**Relative expression level of up-regulated transcription factors (TFs) under sulfate starvation**. Transcription factors were up-regulated more than 2 times (16 genes) under sulfate deficient conditions in both experimental replicates. The up-regulated transcription factors were classified into the same 6 clusters as Figure 3 (class I–V, and others) according to responsiveness to sulfate resupply as indicated by fold changes (>1, upwards arrow; <1, downwards arrow) in transcript accumulations, 30′ (30 min) S/-S, 3 h S/-S and 3 h S/30′ S (Supplemental Table SIII). Fold changes relative to the nitrate- and phosphate-sufficient control are shown. -N; data are from Scheible et al. (2004). -P; Morcuende et al. (2007). Values and colors are in log2 scale.

**Figure 6:**
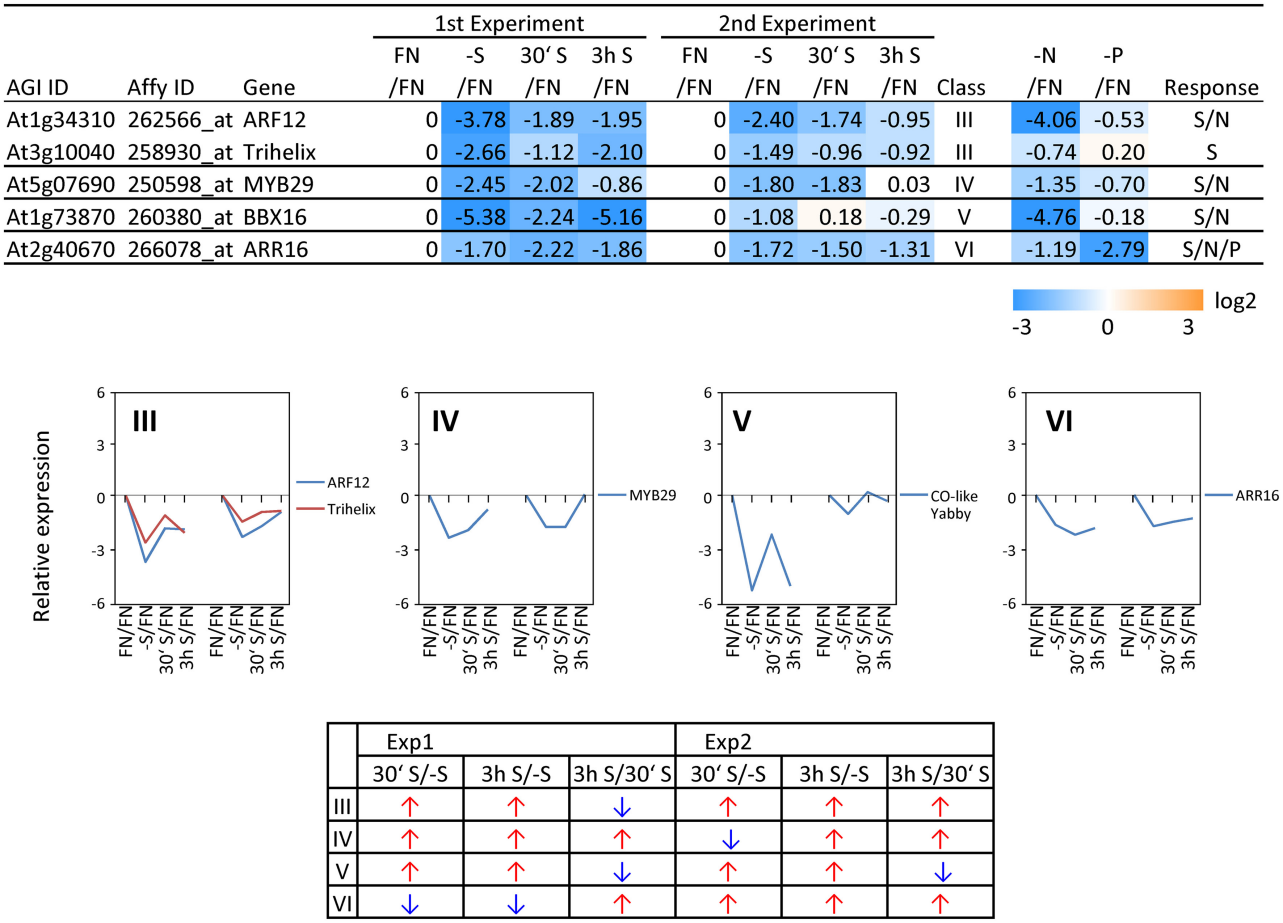
**Relative expression level of down-regulated transcription factors (TFs) under sulfate starvation**. Transcription factors were down-regulated more than 2 times (5 genes) under sulfate deficient conditions in both experimental replicates. The down-regulated transcription factors were classified into the same 7 clusters as Figure 4 (class I–VI, and others) according to responsiveness to sulfate resupply as indicated by fold changes (>1, upwards arrow; <1, downwards arrow) in transcript accumulations, 30′ (30 min) S/-S, 3 h S/-S and 3 h S/30′ S (Supplemental Table SIII). Fold changes relative to the nitrate- and phosphate-sufficient control are shown. -N; data are from Scheible et al. (2004). -P; Morcuende et al. (2007). Values and colors are in log2 scale.

In order to complement the previous transcriptomic studies on sulfur starvation (Hirai et al., 2003; Maruyama-Nakashita et al., 2003; Nikiforova et al., 2003), a wider screen to identify candidate genes regulated by changes in sulfate availability was performed using Affymetrix ATH1 arrays for expression profiling (Supplemental Table SI). Transcript levels of 22746 genes were determined, resulting in eight datasets coming from two biological replicates (four time points each). Depending on the experiment and conditions, 25–36% of the genes were called “absent” by the Affymetrix microarray suite software (MAS version 5.0) (Supplemental Table SII). The changes between transcript levels under FN and sulfate starved conditions were analyzed by calculating the ratio of gene expression levels (-S/FN). To identify up-regulated genes, the genes with “present” and “marginal” calls under -S conditions were used in the comparison -S/FN. Relative expression levels of 55 genes (>5-fold) or 224 genes (>2-fold) were up-regulated and denoted as sulfate starvation responsive genes in both experimental replicates (Figures 2A,C). To identify down-regulated genes, the genes with “present” and “marginal” calls under FN condition were used in the comparison -S/FN. The relative expression levels of 19 genes (<0.2-fold) or 73 genes (<0.5-fold) were down-regulated and grouped as sulfate starvation responsive genes in both experimental replicates (Figures 2B,D). Genes up-regulated more than 5 times (55 genes) were further classified into 6 clusters (class I–V, and others) according to responsiveness to sulfate resupply as indicated by fold changes in transcript accumulations, 30' (30 min) S/-S, 3 h S/-S and 3 h S/30' S (Figure 3) (Supplemental Table SIII). Genes down-regulated more than 5 times (19 genes) were similarly classified into 7 clusters (class I–VI, and others) (Figure 4) (Supplemental Table SIII). The genes in class I were up- or down-regulated under -S and then inversely down- or up-regulated with re-supply of sulfate in a time-dependent manner in both experiments, suggesting them to be genuine sulfate responsive genes, directly responding to the sulfur status of the plant tissue. The list of up-regulated genes in class I contained, among others, known sulfate starvation marker genes such as the sulfate transporters (*SULTR1;2* and *SULTR4;2*) and the rate limiting sulfate assimilation gene (*APR3*; 5'-adenylylsulfate reductase 3) (Vauclare et al., 2002; Hirai et al., 2003; Maruyama-Nakashita et al., 2003; Nikiforova et al., 2003; Hirai and Saito, 2004). Furthermore, several genes in class I contain the SURE core sequence in their 3-kb upstream promoter sequences (Maruyama-Nakashita et al., 2005), and were previously found to be OAS responsive genes (Hubberten et al., 2012a, b) (Figure 3). Genes involved in glucosinolate metabolism were common in the list of down-regulated genes, and five genes were allocated to class I (Figure 4). When applying a 5-fold cut off in the comparison -S/FN, only two TFs [*MYB52* and *MYB75/PAP1* (*PRODUCTION OF ANTHOCYANIN PIGMENT 1*)] were identified in the list of up-regulated genes (Figure 3). Therefore, a 2-fold cut off was applied for identification of candidate TFs (Figures 2E,F) among the approximately 1600 TFs in Arabidopsis (Riechmann et al., 2000; Czechowski et al., 2004, 2005). This yielded 16 genes with relative expression levels >2-fold and five genes with relative expression levels <0.5-fold in the comparison -S/FN in both experimental replicates (Figures 2E,F). These TFs are putatively sulfate starvation responsive TFs. Using the same criteria as applied for Figures 3, 4, the up- or down-regulated TFs were clustered (Figures 5, 6). Among the up-regulated 16 TF genes (>2-fold) under sulfate starvation, the most abundant group was the MYB family with nine genes (Figure 5). Of the up-regulated 16 TF genes, expression of two genes (*MADS*; *At4g33960* and *HAT14* (*HB*); *At5g06710*) responded to sulfate re-supply with repression in both experiments (Figure 5, class I). Among the five TF genes down-regulated (<0.5-fold) under sulfate starvation, none responded to sulfate re-supply with induction in both experiments, but rather stayed repressed within the time frame studied (Figure 6).

### Reproducibility between 1st and 2nd experiments

The reproducibility of all measurements was checked by simultaneous analysis of two experimental replicates (experiment 1 and 2). In both experiments, plants were grown in exactly the same way using the same stock of Col-0 seeds. However, numbers of up- or down-regulated genes and the magnitude of changes were higher in experiment 1 than experiment 2 (Figure 2). Such variability seems to be reasonable, when considering experiment 1 was more severe in terms of sulfate starvation, e.g., lower sulfate and GSH contents under the -S condition as compared to experiment 2 (Figure 1). In addition to the decrease of sulfate content, nitrate content was also decreased under -S conditions in experiment 1 (Supplemental Figure S1). Phosphate content was increased in experiment 1 and was not changed in experiment 2 (Table 1). Several genes, including TFs, which specifically changed in the –S condition in experiment 1 also responded in nitrate starvation conditions (Supplemental Table SIV). Thus, we took the genes in the intersection of both experiments to further investigate sulfur responsive genes (Figure 2).

### Sulfur-specificity of candidate TF genes

To examine the sulfur-status specificity of the selected TFs, their expression in response to nitrate and phosphate starvation was analyzed (Figures 5, 6). Both nutrient-stress experiments (nitrate and phosphate) were performed using the same culture system, including the same light conditions in the same phytotron chamber, with the same basic media, except for differences in a single nutrient (Scheible et al., 2004; Morcuende et al., 2007). Of the 21 sulfate starvation responsive TFs, 18 also responded to other stimuli besides sulfate starvation, indicating that they are parts of more general nutrient depletion response modules. Such interconnectivity between various nutrients has been shown previously (Watanabe et al., 2010). However, the strength of the response or the direction of the response, whether up- or down-regulated, varies between different nutrient starvation conditions. This must require specific upstream regulatory elements governing the individual distinct response schemes of these TFs for the respective nutrient depletion conditions. However, four up-regulated TF genes (*MADS*, *HAT14*, *MYB31* and *MYB45*) and one down-regulated TF (*Trihelix*; *At3g10040*), which we speculated to be genuine sulfur status responsive genes among plant nutrient responses, were neither induced under nitrate nor phosphate starvation with a 2-fold cut off, suggesting that these four genes might be specific for the regulation of the response to sulfate starvation. Interestingly up- or down-regulated class I genes showed fewer responses to nitrate and phosphate starvations compared to the genes in other classes (Figures 3, 4). The comparison with nitrate and phosphate starvation resulted in the identification of additional genes that generally responded to mineral nutrient stresses (N, P, and S). These genes were *MYB75/PAP1*, *MYB52*, and *ATAF1/NAC02*, *DREB A-4* (Figure 5) and *ARR16* (Figure 6). Nine genes (*MYB54*, *MYB9*, *MYB53*,* MYB71*,* MYB93*,* WRKY56*,* ARF12*,* MYB29*, and *BBX16*) showed an overlap between N and S starvation and did not respond to P starvation, while only two genes showed a direct co-behavior to P starvation not shared by -N conditions (*ZAT6* and *ZAT12*) (Figures 5, 6).

### Expression changes downstream of known transcription factors

**Figure 7:**
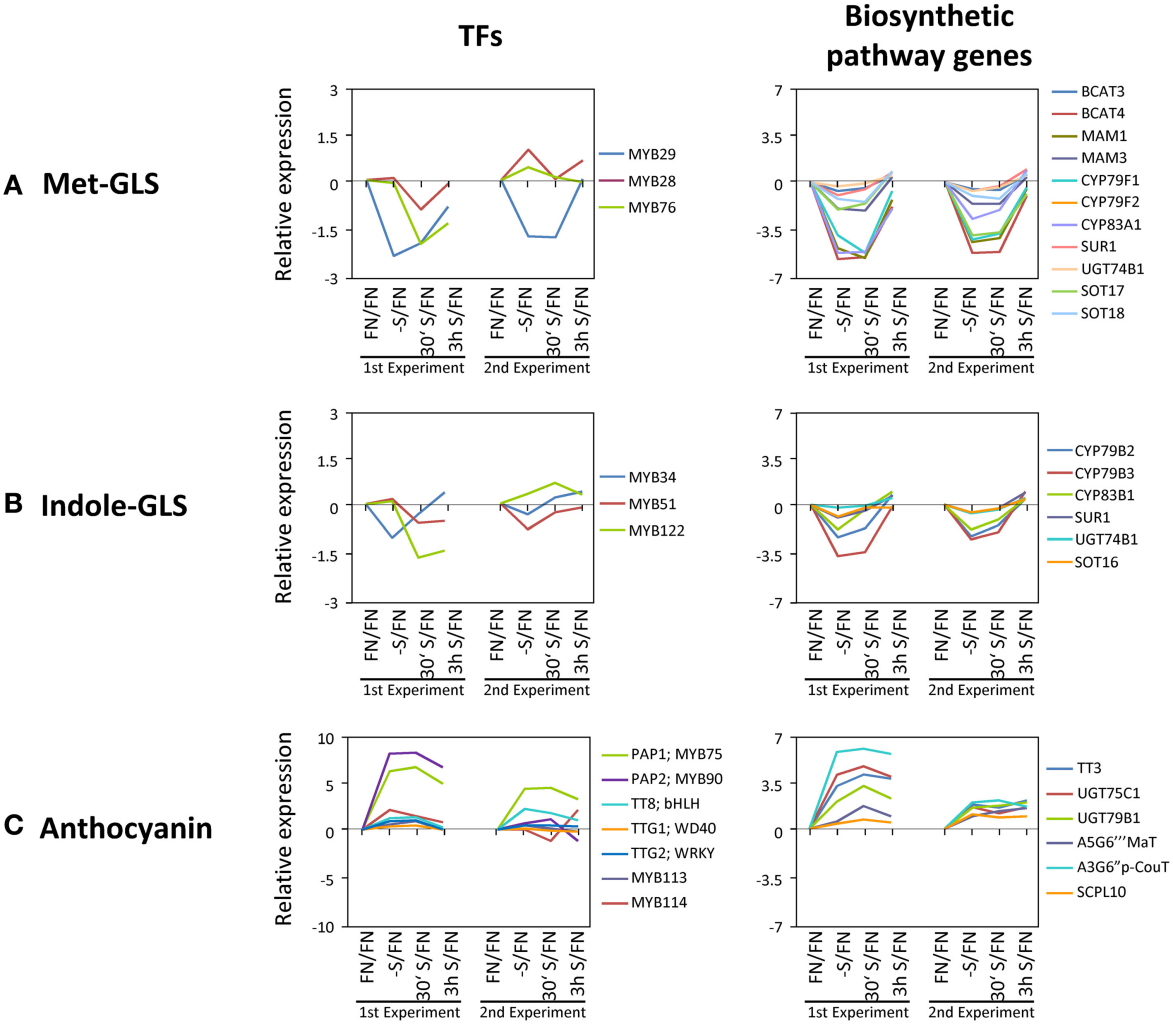
**Figure 7. Gene expression changes in known transcription factors and the downstream genes. (A)** Methionine derived glucosinolates (Met-GLSs). **(B)** Tryptophan derived glucosinolates (Indole-GLSs). **(C)** Anthocyanin AGI IDs and full names of genes are in Supplemental Table SV. Values are in log2 scale.

*MYB75/PAP1* expression was up-regulated (Figures 3, 5) and *MYB29* down-regulated (Figure 6) under sulfate starvation. These genes were reported to be positive regulators of anthocyanin production and glucosinolate production, respectively (Borevitz et al., 2000; Hirai et al., 2007). Additional TFs were reported to be involved in regulating anthocyanin production (*MYB90/PAP2*, *MYB113*, and *MYB114* for anthocyanin, *TT8* (*TRANSPARENT TESTA8*); *bHLH*, *TTG1* (*TRANSPARENT TESTA GLABRA1*); *WD40* and *TTG2* (*TRANSPARENT TESTA GLABRA2*); *WRKY* for flavonoids) (Tohge et al., 2013) and glucosinolate production [*MYB28* and *MYB76* for methionine derived glucosinolates (Met-GLSs), and *MYB34*, *MYB51*, and *MYB122* for tryptophan derived glucosinolates (indole-GLSs)] (Celenza et al., 2005; Hirai et al., 2007; Sonderby et al., 2007; Gigolashvili et al., 2007a, b, 2008; Malitsky et al., 2008). We investigated whether these TFs were responding to sulfate starvation similarly to *MYB75/PAP1* and *MYB29*, and we further screened whether the respective downstream target genes of the biosynthetic pathways showed any correlation in expression characteristics (Figure 7). For this, we additionally included values with “absent” calls. Biosynthetic genes, which are involved in production of both Met-GLSs and indole-GLSs were down regulated under –S conditions and re-induced under re-supply of sulfate (Figure 4). The gene expression patterns of the biosynthetic genes specifically followed that of *MYB29* (Figure 7A). *MYB34* also showed a similar pattern to the biosynthetic genes, but with smaller changes in expression (Figure 7B). These results suggest that *MYB29* and *MYB34* seem to be major regulators under sulfate starvation for Met-GLSs and indole-GLSs, respectively. *MYB75/PAP1* was an up-regulated gene and clustered to class IV, which showed a slow tendency for being repressed with re-supply of sulfate (Figure 3). The gene expression patterns of anthocyanin biosynthetic genes and other TFs followed that of *PAP1* in experiment 1, although other TFs, except for *PAP2*, showed changes lower than the previously applied threshold (Figure 7C). In experiment 2, biosynthetic genes showed smaller changes in expression compared to experiment 1 and mixed patterns, which might be caused by the mixed patterns of other TFs such as *PAP2*, *TT8*, and *MYB114*.

## Discussion

### Molecular and physiological responses to sulfate starvation and replenishment revealed a systemic internal rebalancing of plant metabolism

In order to investigate early changes in the transcriptome in response to sulfate starvation and consecutive replenishment, Arabidopsis seedlings were subjected to a short-term sulfate starvation followed by short-term re-supply of 30 min and 3 h. Axenic, 10-days old seedlings were transferred to sulfate-free conditions for 48 h. Under these conditions, Arabidopsis seedlings did not exhibit the typical phenology of sulfate limited plants, which are known to be: reduced chlorophyll, accumulation of anthocyanins in the leaves, and altered root growth (Nikiforova et al., 2003; Hubberten et al., 2012a). In contrast, Arabidopsis seedlings deprived for nitrate or phosphate, showed typical phenotypic responses (Scheible et al., 2004; Morcuende et al., 2007). Although of key importance for plant metabolism, abundance of sulfur is about 7% that of nitrogen in shoot tissues (Buchanan et al., 2000). Hence, the observed differences in starvation symptoms become reasonable as severe effects develop slower.

### Sulfate starvation leads to induction of sulfate response genes which results in an overshoot of sulfur containing metabolites prior to sulfate accumulation

Observations of changes in metabolite levels under short-term sulfate starvation and after sulfate re-supply provide novel information as only the metabolome under prolonged sulfate starvation has been previously described (Nikiforova et al., 2005b). Metabolites from the primary sulfate assimilation pathway such as Cys and GSH are reduced to very low levels under sulfate starvation, similarly to sulfate (Figure 1) and plant total sulfur (Supplemental Figure S2). This indicates that the plants were exposed to sulfate starvation that depleted the internal stores. Cys, GEC, and GSH rapidly respond to resupply of sulfate. After 30 min of sulfate re-supply their pools already started to be restored and in the case of Cys and GEC, the levels exceeded the initial status of plants under FN up to 2-fold (Cys) after 3 h re-supply. This suggests that the re-supplied sulfate is immediately used for synthesis of Cys and GSH instead of accumulation of excess sulfate in the vacuole, as the sulfate levels only slightly increase after 3 h. In fact, the uptake and assimilation pathways have been reported to be induced and act efficiently at utilizing re-supplied sulfate in barley (*Hordeum vulgare* L.) (Smith et al., 1997) and curly kale (*Brassica oleracea* L.) (Koralewska et al., 2009). However, none of the downstream metabolites such as homocysteine, methionine or putrescine return to the control levels, even after 3 h; total sulfur levels are also not substantially increased (Figure 1, Supplemental Figure S2 and Table 1). The overshoot of Cys indicates firstly the increased capacity of the uptake and assimilation system to utilize sulfate as corroborated by the expression of high affinity sulfate transporters (*SULTR1;1* and *SULTR1;2*) and the induction of *APR3* (Koralewska et al., 2009). Secondly, the early accumulation of GEC prior to GSH might indicate a slow interconversion between the subcellularly separated metabolite pools, where GEC is exclusively synthesized in the chloroplasts, while GSH is synthesized both in the cytosol and the chloroplast (Noctor et al., 2002). GEC biosynthesis appears to be down-regulated only when GSH, itself a potential regulator, has accumulated to control cellular levels. Interestingly, this is not accompanied by changes in expression of the *GSH1* and *GSH2* genes. Thirdly, after 3 h of replenishment plant homeostasis has not returned to control levels as, for example, amino acid pools remain disturbed. The continued reduction of the total sulfur content indicates that as yet insufficient amounts of sulfate have been taken up and assimilated to (i) replenish the depleted metabolite pools, such as glucosinolates, proteins and others and (ii) to allow sequestration of excess sulfate to the vacuole.

This response is quite similar to the response of plants exposed to phosphate starvation and re-supply (Morcuende et al., 2007). Phosphate starvation induces numerous genes involved in phosphate uptake and assimilation, additionally catabolic reactions are induced. Upon phosphate re-supply the content of free phosphate does not increase immediately but accumulates slowly while various phosphorus containing metabolites, such as the central metabolites glucose-6-phosphate and ATP (adenosine triphosphate), recover faster. Thus, this response is comparable to the response we observe for sulfate starvation. The uptake and assimilation systems are induced by depletion which results in a fast conversion of any available nutrient ion into the organic fraction which rises before free nutrient ions can accumulate.

Nitrate starvation shows a quite different behavior toward starvation (Scheible et al., 2004; Konishi and Yanagisawa, 2014). Here nitrate-depletion rather reduces part of the nitrate uptake and assimilation machinery. Nitrate availability then induces genes involved in uptake and assimilation of nitrate (Wang et al., 2004). Hence, nitrate accumulates first in leaf tissues upon re-supply after depletion and successively organic nitrogen compounds accumulate (Scheible et al., 1997). Despite the similarity between nitrate and sulfate assimilatory pathways (Hesse et al., 2004c), their regulation in response to availability of the respective nutrient ions is different.

### Effect of sulfate starvation on polyamine metabolism

Upon sulfate starvation, putrescine accumulates in plant tissues. In parallel it may be assumed that SAM levels are reduced (Nikiforova et al., 2005b), impeding the biosynthesis of the downstream polyamines spermine and spermidine, with the effect that the co-substrate putrescine accumulates. One of the spermine synthases, *At5g53120*, is induced to favor this biosynthetic route. Among the class I response genes, arginine decarboxylase (*ADC*; *At4g34710*) is induced (>5-fold) (Figure 3), as well as agmatinase (*At4g08870*; the latter only in experiment 1; >2-fold), and which synthesizes putrescine, releasing urea; both are likely to contributie to the observed accumulation of putrescine (Table 1). On the other hand, the alternative pathway via agmatine iminohydrolase, shows repression of the respective gene (*At5g08170*). Agmatinase releases urea, which has been shown to accumulate under sulfate starvation (Nikiforova et al., 2005b), while agmatine iminohydrolase releases ammonium. Sulfate starved plants show accumulation of nitrogenous compounds and therefore it would be sensible to prevent the accumulation of toxic ammonium concentrations. As *ADC* is part of the class I response gene category and in fact is induced under sulfate starvation, but not nitrate and phosphate (Figure 3), it might be possible that members of the class I TF genes are regulating those genes involved in polyamine biosynthesis under sulfate starvation thus providing a testable hypothesis.

### Effect of sulfate starvation on vitamin E metabolism

Metabolite profiling in this study further revealed new sulfur starvation responsive metabolites, which could be involved in plant strategies to alleviate sulfate starvation stress. Interestingly, two metabolites from the vitamin E biosynthesis pathway were changed under sulfate limited conditions. γ-tocopherol, a direct precursor of α-tocopherol, which is the major vitamin E compound found in leaf chloroplasts (Munne-Bosch, 2005), and it's precursor, 2,3-dimethyl-5-phytylquinol, were both elevated under sulfate starved conditions, when compared to FN conditions in both experiments (Table 1). Tocopherols are presumed to be important antioxidants deactivating photosynthesis-derived reactive oxygen species and preventing the propagation of lipid peroxidation by scavenging lipid peroxyl radicals in thylakoid membranes. It is generally assumed that increases of α-tocopherol contribute to plant stress tolerance, while decreased levels favor oxidative damage. Tocopherols together with other antioxidants such as ascorbate, GSH, carotenoids, isoprenoids, flavonoids, and enzymatic antioxidants ensure the adequate protection to the photosynthetic apparatus and help plants to withstand environmental stress (Hollander-Czytko et al., 2005; Munne-Bosch, 2005). Under conditions of sulfate starvation, GSH levels drop dramatically (Figure 1) (Nikiforova et al., 2005b) and hence ascorbate pools cannot be regenerated. Increased levels of other compounds with antioxidant capacity, e.g., the tocopherols identified in this study, would compensate for this loss. This is supported by the result that tocopherol biosynthetic genes [*VTE* *VITAMIN E DEFICIENT*)-1; *At4g32770* (2.0-fold), −2; *At2g18950* (2.2-fold) and −4; *At1g64970* (3.0-fold)] were induced under sulfate starvation in experiment 1 and *VTE1* (1.8-fold) in experiment 2. In conclusion, the necessity to maintain viability in conditions where sulfur, a crucially important macronutrient, is deficient, results in a systemic internal rebalancing of plant metabolism. This is reflected by decreased or increased levels of distinct metabolites in sulfur-deficient plants. The shift in balance and regulation of metabolism, including re-allocation of compounds, allows the plant to readjust its homeostasis and to remain viable and to still be able to produce seeds for dispersal, despite the adverse conditions.

### Transcriptomics provides information on candidate transcription factors regulating S metabolism

Sulfate starvation leads to increases in the expression of sulfate-responsive genes, as reviewed by Hirai and Saito (2004) and Nikiforova et al. (2004), Nikiforova et al. (2005a) and OAS responsive genes (Hubberten et al., 2012a, b). When applying whole-genome chips for expression studies of sulfate starvation we identified novel candidate TFs which have not been seen in previous studies. Additionally, through classification into response groups and correlation, we could derive information on transcriptional co-behavior between sulfur starvation responsive genes (Figures 3, 4, 6) and TFs (Figure 5). This allowed further monitoring of the expressional changes for whole biosynthetic pathways (Supplemental Table SI). This will facilitate drawing of working hypotheses concerning regulatory circuits, especially if this is further corroborated by metabolite data.

By applying a set of selection criteria such as response to (i) sulfate starvation, (ii) re-supply of sulfate, and (iii) other nutrient starvations (nitrate and phosphate), we identified 21 sulfate starvation responsive TF genes which were categorized to be specifically S-starvation responsive, generally responsive to S, N and P-depletion, or to S and N, or S and P-deprivation (Figures 5, 6). The function of five S-specific TFs *MADS*, *HAT14*, *MYB31*, *MYB45* (up-regulated; Figure 5), and *Trihelix* (down-regulated; Figure 6) is still completely unknown. We suggest that these TFs are involved in regulating S-metabolism under varied sulfate availability to the plant, and it is thus possible that sulfate responsive class I genes are putative targets based on their temporal co-expression pattern (Figure 3; class I).

The five general (S/N/P) TFs have been studied previously, especially *MYB75/PAP1* as a positive regulator for anthocyanin production, under various abiotic stress conditions (Borevitz et al., 2000). *ATAF1* (Figure 5) is a *NAC* gene and has been reported to be involved in plant adaptation to abiotic and biotic stresses and development (Wang et al., 2009; Wu et al., 2009). *ATAF1* activates ABA (abscisic acid) biosynthesis, which is critical for plant stress responses (Jensen et al., 2013). *ARR16* (Figure 5) has been reported to be a response regulator which is involved in the cytokinin signaling pathway mediated by AHK4 (histidine kinase) in roots (Kiba et al., 2002). Cytokinins are a class of plant hormones important for the regulation of cell division and differentiation (Mok and Mok, 2001). Several reports have implicated cytokinins in responses related to the status of nutrients such as sugar, nitrogen, phosphorus, and sulfur (for review, see Franco-Zorrilla et al., 2004; Maruyama-Nakashita et al., 2004; Sakakibara et al., 2006). *MYB52* (Figure 5) is one of the SND1 (SECONDARY WALL-ASSOCIATED NAC DOMAIN PROTEIN1)-regulated TFs, suggested to be involved in the regulation of secondary cell wall biosynthesis (Zhong et al., 2008). Secondary cell walls provide mechanical strength and facilitate the transport of water and nutrients (Wang and Dixon, 2012). *MYB75/PAP1* has been also reported to be involved in regulation of secondary cell wall formation as a repressor of the lignin branch of the phenylpropanoid pathway (Bhargava et al., 2010). Interestingly, the *CSLG3* gene (cellulose synthase-like protein; *At4g24990*) (Figure 3; class I) is highly co-expressed with *MYB75/PAP1* (ATTEDII; Obayashi et al., 2007).

One of the S/N-TFs, *MYB54* (Figure 5), is also a SND1-regulated TF like *MYB52*, suggested to be a regulator for secondary cell wall biosynthesis (Zhong et al., 2008). Another S/N-TF, *MYB93* (Figure 5), has been reported to be a negative regulator of lateral root development (Gibbs et al., 2014). *MYB93* is part of a novel auxin-induced negative feedback loop stimulated in the endodermis upon lateral root initiation to ensure that lateral roots are formed only in the correct place (Gibbs et al., 2014). The S/N-TF *MYB53* (Figure 5) is a member of a small subfamily of Arabidopsis R2R3 MYB TFs, which also contains *MYB93* and *MYB52* (Kranz et al., 1998; Stracke et al., 2001), suggesting a similar function of *MYB53* to *MYB93* for lateral root initiation. Auxin represents a key regulator of lateral root development (Blakely et al., 1982; Laskowski et al., 1995). The S/N-TF *ARF12* (Figure 6) is an auxin response factor and showed a root cap-specific expression (Rademacher et al., 2011), suggesting a role of *ARF12* in regulation of root development under stress conditions. Further, the involvement of auxin related TFs, which were identified as potential transcriptional regulators of sulfur metabolism by a systems approach (multifactorial correlation network) using sulfur starvation experiments (Nikiforova et al., 2005a), has been associated to the sulfate starvation response (Falkenberg et al., 2008).

The B-box (BBX) proteins are a class of zinc-finger TFs containing a BBX domain with one or two BBX motifs. BBX proteins control growth and developmental processes that include seedling photomorphogenesis, photoperiodic regulation of flowering, shade avoidance, and responses to biotic and abiotic stresses (Gangappa and Botto, 2014). Some members of the BBX family (BBX21, 22, 24, and 25) have been reported to interact with HY5 (ELONGATED HYPOCOTYL5), which is a basic domain/leucine zipper (bZIP) TF, central for the regulation of seedling photomorphogenesis (Gangappa and Botto, 2014). HY5 plays an important role in regulation of sulfate assimilation through the regulation of *APR* gene expression (Lee et al., 2011). The S/N-TF *BBX16* (Figure 6) has been reported to promote branching and to suppress hypocotyl elongation (Wang et al., 2013). Among the two S/P-responsive TFs, *ZAT6* has been reported to regulate root development and nutrient stress responses, especially phosphate starvation (Devaiah et al., 2007), to which we can add a putative function in –S response. Induction of *ZAT6* under phosphate starvation leads to a decrease in the primary root growth, but increases the root-to-shoot ratio by promoting lateral root growth. *ZAT12* has been implicated in multiple abiotic stress responses such as high light (Demmigadams and Adams, 1992; Iida et al., 2000), wounding (Chen et al., 2002; Cheong et al., 2002; Rizhsky et al., 2004), low-oxygen (Klok et al., 2002), hydrogen peroxide (Desikan et al., 2001), heat, treatment with paraquat (Rizhsky et al., 2004), and cold (Wise and Naylor, 1987). As these stresses are associated with the formation of reactive oxygen species, the role of the *ZAT12* may be to help plants cope with oxidative stress (Davletova et al., 2005; Vogel et al., 2005). Interestingly, *ZAT12* over-expression resulted in accumulation of transcripts encoding arginine decarboxylase (*ADC*; *At4g34710*) (Vogel et al., 2005). Both *ZAT12* and *ADC*, were induced under sulfate starvation (Figures 3, 5), suggesting that *ZAT12* is a possible positive regulator of *ADC* and hence for polyamine production. Polyamines have been shown to have protective roles against abiotic stress, including oxidative stress (Ye et al., 1997; Bouchereau et al., 1999). The functions of the genes *DREB A-4* (S/N/P), *MYB9*, *MYB71*, and *WRKY56* (S/N) (Figure 5) have not been identified yet, but it can be assumed that they are also involved in the response to plant nutrient deprivation.

The majority of the sulfate starvation-regulated TFs also responded to other nutrient depletions, however, the levels of expression were different indicating specific response schemes for different stresses (Figures 5, 6). This suggests the involvement of these TFs in multifactorial response networks (Broun, 2004) and that the plant uses certain gene sets or modules for various purposes (Watanabe et al., 2010). There was a greater overlap between TF genes induced by nitrate and sulfate than sulfate and phosphate. It has been previously shown that nitrogen and sulfur metabolism are linked closely (Hesse et al., 2004b), and that amino acid homeostasis in particular, to which both nutrients contribute, is a key feature of plant metabolism (Hofgen et al., 1995; Nikiforova et al., 2006). The existence of such a network of common nutrient-responses might explain the fact that TF genes positively and negatively induced by sulfate starvation, exhibited the same tendency with regard to nitrate and phosphate limitations, with only a few exceptions (Figures 5, 6).

## Conclusions

This transcriptome and metabolome study on the response of *Arabidopsis thaliana* seedlings toward sulfate depletion and replenishment, in conjunction with previous data on phosphate and nitrate treatments, respectively (Scheible et al., 2004; Morcuende et al., 2007) provided novel information with respect to genes and metabolites involved in the efforts of the plants to retain homeostasis while a single macronutrient, here sulfate, is depleted. Co-response behavior analysis allowed grouping the data set into concise classes of common response behavior. This provides a basis for future analysis of the respective genes and assigning functions to as yet uncharacterized genes. As an example, to further our knowledge on the regulation of the underlying processes, we extracted the TFs from the data. Categorization again allowed assignment, and thus putatively linking them functionally, to the gene response classes. Moreover, comparison to the data sets from similar nitrate and phosphate treatments allowed scoring for either specific or shared TFs between distinct mineral nutrient depletions. For nutrient stress response networks we can thus conclude that the plant recruits common stress response networks including jointly utilized TFs, but provides necessary specificity through a subset of specific TFs. Furthermore, we were able to link some of the response chains, i.e., TF/structural genes/metabolite response, to the physiological response of the plant system (Figures 6, 7). One example is that *MYB29*, a TF controlling glucosinolate biosynthetic genes, is down-regulated upon sulfate starvation and de-repressed upon sulfate re-supply; several glucosinolate biosynthetic genes follow this behavior and as a result the amount of glucosinolates, which are sulfur-rich secondary compounds in brassicaceae, is reduced, and respectively recovers after replenishment, which physiologically helps the plant to save sulfate resources for primary metabolism under sulfate deprived conditions. We assume that the data set can be mined for further response modules.

## Comment

Systems analysis of plants exposed to varied mineral nutrient availability provides a plethora of molecular information. The main objective is to filter relevant information from noise. With respect to transcriptomics this can, as shown in this study, be achieved through time resolved sampling which generates kinetic information on the gradual development of an expression response. When employing arrays or chip based technologies this might provide insufficient or ambiguous information especially with respect to low expressed genes which might result in “absent calls.” More sensitive methods such as quantitative real time RT-PCR using e.g., extended primer platforms (Udvardi et al., 2008) might close this gap. Next generation sequencing further facilitates increasing the dynamic range of transcriptome studies by obtaining quantitative information on high to very low expressed genes, given that a sufficient coverage can be achieved. To analyze and filter these data an effective and thorough statistical analysis is necessary to evaluate the data. Suitable software is made available through various sources, e.g., on www.mpimp-golm.mpg.de. Specific transcriptome data e.g., using polysome bound RNA (Piques et al., 2009) provides valuable information with respect to the translatome and which expressed genes are indeed converted to protein. A necessary step will be a more specific sampling at the tissue level, not only differentiating between gross structures such as roots, shoots and flowers, but targeting substructures. Such efforts have been made for Arabidopsis roots (Iyer-Pascuzzi et al., 2011), for potato guard cells (Plesch et al., 2001) and for Medicago arbuscules (Gaude et al., 2012), but need to be expanded in order to understand the interplay of the various distinct specialized tissues. However, transcriptomics usually provides only information of differentially expressed genes and needs to be complemented by mutational approaches, GWAS, or to be approached by complementary technologies such as protein profiling.

Metabolomics provides information on the metabolic status of a system, which however is clock dependent, modulated by environmental conditions, and subject to developmental changes. It provides information for when a system deviates from controls upon stress, such as sulfate starvation. This might indicate affected pathways for further scrutinization or help corroborate transcriptome data. An important contribution toward understanding resource allocation and it's regulation will be flux studies by e.g., using specific isotopes (Giavalisco et al., 2011). It may be considered that the plant system tries to attain a homeostatic state, thus, metabolome data as the integrative readout of transcript alterations and changes of enzyme activities and abundances, respond with damped signals, unless severe stresses or mutations lead to massive homeostatic shifts.

To complete the systems analysis, proteome data, enzyme activity data, and protein-protein interactomes from similar tissue samples as above should be obtained. Such a comprehensive dataset will allow creation of multifactorial networks as wiring schemes of plant metabolism and physiology. Eventually, bioinformatics should aim at modeling these complex networks, identify their modular components and capture the flexibility of the system (Nikiforova et al., 2005a, b; Watanabe et al., 2010) and facilitate predictions (Hansen et al., 2014). Bioinformatics approaches are available to, for example, extract context-specific metabolic networks from transcriptome data, which will help the understanding of the features of certain sub-networks or modules (Becker and Palsson, 2008; Estévez and Nikoloski, 2014). The difficulty is the inherent flexibility of multifactorial networks, which is the basis of plant adaptive flexibility and, furthermore, the enormous biodiversity between plant species, even cultivars. Finally, insufficient computing power to analyze and evaluate these enormous databases restricts these approaches, limiting analyses to data subsets. The development and refinement of ‘omics tools allows, especially with respect to plant nutrition, the description of multifactorial systems. This will allow *in silico* predictions of key components, which will need to be verified in an iterative way using mutants or reverse genetics, before eventually exploiting knowledge for crop breeding and improvement.

## Materials and methods

### Plant material

In all experiments the *Arabidopsis thaliana* (L.) ecotype Col-0 was used.

### Seed sterilization

Seeds were sterilized by adding 70% ethanol for 2 min and 3% sodium hypochloride (NaClO) with one drop of Triton X100 for next 15 min. The NaClO/Triton X100 solution was removed and seeds were washed with sterile water 3–5 times. After removing the water seeds were resuspended in 0.1% sterile agarose for imbibition.

### Sterile liquid cultures

Wild-type Col-0 seedlings (100–120 seeds) were grown in 30 ml of sterile liquid FN medium or 150 μ M SO_4_^2−^ medium (250 mL Erlenmeyer glass flasks) on orbital shakers with constant, uniform fluorescent light (approximately 50 μE in the flask) and temperature (22°C). Shaker speed was low (30 rpm) during the first 3 days, and then increased to 80 rpm. Care was taken to prevent significant clumping of seedlings. After 7 days the FN media was replaced with another 30 ml of fresh FN medium, whereas the 150 μ M SO_4_^2−^ medium was replaced with 30 ml of low sulfate (-S) medium, in which plants were subjected for sulfur starvation for 2 days. On day 9 FN cultures and some of the -S cultures were harvested. At the same time all other flasks of S-starved cultures were opened, and re-closed either without supply or after supply of 1 mL 15 mM K_2_SO_4_ (500 μ M final concentration) or 1 mL 15 mM KCl (500 μ M, control). Cultures re-supplied with sulfate ions (or KCl) were harvested after 30 min and 3 h. Plant material from each flask was quickly blotted on tissue paper, washed twice in an excess of deionized water, blotted on tissue paper again and frozen in liquid nitrogen. Materials were stored in liquid nitrogen until pulverization using mortar and pestle. Ground material was stored at −80° C until further use. Medium compositions (FN, 150 μ M SO_4_^2−^ and low S) are in Supplemental Table SVI.

### RNA extraction using TRIzol mini-prep protocol

Frozen plant material (100–200 mg) was ground in liquid nitrogen in a pre-cooled mortar or homogenized using metal beads (Ø 5 mm) in the MM200 homogenizer (Retsch). TRIzol reagent (Invitrogen) was then added and mixed well by vortexing. After 5 min incubation at room temperature the homogenate was centrifuged at 13,000 × g for 5 min at 4°C in pre-cooled centrifuge. The supernatant was removed to a fresh Eppendorf tube and 400 μL of chloroform was added and mixed by vortexing before incubation at RT for 5 min. After 10 min centrifugation at 13,000 × g, at 4°C, the aqueous phase (approximately 1 mL) was transferred to a fresh Eppendorf tube. RNA was precipitated with 0.5 mL of isopropanol and 0.5 mL of HSS buffer (0.8 M sodium citrate and 1.2 M NaCl) per 1 mL of aqueous phase, overnight at −20°C. The precipitate was pelleted by centrifugation at 13,000 × g for 30 min at 4°C. The supernatant was removed and the pellet was washed twice with 1 mL 70% ethanol, air dried, and re-suspended in ca. 50 μL water (approximately 1 μg RNA/1 μL). RNA concentration, purity and integrity were determined by analysis with Bioanalyser (Agilent) according to the manufacturer's instruction.

### Transcription profiling on full genome chip

ATH1 (Affymetrix, 22,800 genes of *Arabidopsis thaliana*, each represented by 11 oligonucleotides and mismatches) was used for transcript level observation in order to perform a genome-wide analysis. For hybridization of the ATH1 chips, 50 μg of clean and intact RNA from each sample of sterile liquid cultures plant material was sent together with the ATH1chips to German Resource Center for Genome Research (RZPD, Berlin, Germany). The data discussed in this publication have been deposited in NCBI's Gene Expression Omnibus (Edgar et al., 2002) and are accessible through GEO Series accession number GSE64972.

### Measurement of thiols

Around 100 mg of the powdered frozen plant material was used for the 1:5 extractions of thiols with 0.1 M HCl. Thirty mg of polyvinylpolypyrrolidone (PVPP) was added immediately to the extraction mixture. The PVPP had been washed before with 0.1 N HCl and dried. The extraction was done under shaking with 500 rpm for 40 min at RT. After centrifugation for 15 min at 4°C and 14000 rpm the supernatant was removed and either analyzed immediately or stored at −20°C. Extracted thiols were first transferred quantitatively into their reduced form. Reduction step was implemented with the addition of 70 μl freshly prepared 10 mM DTT to 120 μl of the extract with 200 μL of 0.25 M *N*-cyclohexyl-2-aminoethanesulphonic acid (CHES) buffer (pH 9.4 with sodium hydroxide [NaOH]). The sample mix was incubated for 40 min at RT. The reduced thiols were transferred into fluorescently active derivatives, by the fluorescent dye monobromobimane (mBrB, 3-bromomethyl-5-ethyl-2,6-dimethyl-pyrazolo[1,2-α]pyrazol-1,7-dione, Calbiochem). Derivatization was carried out during incubation with 10 μL 25 mM mBrB in acetonitrile for 15 min at RT in the dark. The reaction was stopped by the addition of 220 μL 15% HCl and incubation for 30 min at 4°C in the dark. After spinning down the cell debris for 20 min at 4°C and 14000 rpm, the supernatant was transferred into HPLC glass vials with lids and either stored at 4°C in the dark or directly measured. Labeled thiols were separated chromatographically by reversed phase chromatography (RP-HPLC) [according to Fahey et al. (1981)] and sensed by a fluorescent detector. 30 μl of derivatized extract was applied to the octadecyl silicate column (25 cm in length and 4 mm in diameter with a grain size of 5 μm, Knauer) and separated by an increasing gradient of hydrophobicity (Supplemental Table SVII) with a flow rate of 1 mL/min. The hydrophobicity gradient was obtained by mixing two different elution solvents (Supplemental Table SVII). The fluorescence measurement of mBrB derivatives occurred at 480 nm emission wavelength under light excitation of 380 nm.

### Measurement of free amino acids

Amino acids were derivatized with ortho-phthaldialdehyde (OPA), a fluorescence dye (Lindroth and Mopper, 1979; Kreft et al., 2003). Amino acids were extracted through a multiple step procedure with *N*-(2-hydroxyethyl)-piperazine-*N'*-(2-ethanesulphonic) acid (HEPES) and different ethanol concentrations [according to Scheible et al. (1997)]. Four hundred μL 80% ethanol in 2.5 mM HEPES (pH 7.5 with KOH) were added to 100 mg of ground frozen plant material and the mixture was shaken for 20 min at 80°C with 500 rpm. After centrifugation for 10 min at 4°C and 14000 rpm the supernatant was removed and re-extracted with 400 μL 50% ethanol in 2.5 mM HEPES (pH 7.5) and shaken for 20 min at 80°C with 500 rpm. After another centrifugation for 10 min at 4°C and 14000 rpm the supernatant was removed and for a third time extracted with 200 μL 80% ethanol in 2.5 mM HEPES (pH 7.5) again for 20 min at 80°C with 500 rpm. The extraction mix was centrifuged a last time for 10 min at 4°C and 14000 rpm and the supernatant was taken and either immediately analyzed or stored at −20°C. All extracted amino acids were mixed with 0.2 volume with 0.8 M borate buffer (pH 10.4, Crom Analytic) just before the measurement procedure and then filled into the HPLC glass vials with lids. Next 70 μL of the sample volume was mixed in the ratio 1:1 with the OPA-derivation reagent, which was composed of 0.5% (w/v) OPA in 0.7 M borate buffer with 10% (v/v) ethanol and 1% (v/v) β -mercaptoethanol. The incubation time was 1 min, directly performed in the injection sampler to achieve reproducible results. For separation of amino acids, an RP-column, 12 cm in length and 4.6 mm in diameter with a grain size of 3 μm and octadecyl silicate as stationary phase (Knauer), was used. 15 μL of the OPA-labeled sample mix were injected and separated by a non-linear gradient of two different buffers as described in Supplemental Table SVIII. The buffers differed in their hydrophobicity and composition as illustrated in Supplemental Table SVIII and were pumped with a flow rate of 0.8 mL/min. The fluorescence detection of the OPA-labeled amino acid derivatives was detected at 450 nm emission wavelength and an excitation wavelength of 330 nm.

### Measurement of polyamines

RP-HPLC in combination with fluorescence spectrophotometry was used to separate and quantitate polyamines (putrescine, spermidine, and spermine) through their dansyl derivatives (Smith and Davies, 1985). One hundred mg of the powdered frozen plant material was used for the extraction of polyamines with 1 mL of 0.2 M perchloric acid (PCA; HClO_4_). After incubation for 1 h at 4°C the homogenates were centrifuged for 30 min at 4°C and 14000 rpm. The supernatant and pellet were collected separately. The supernatant was used to determine PCA-soluble free (fraction 1) and PCA-soluble conjugated polyamines (fraction 2), whereas the pellet was used to determine PCA-insoluble bound polyamines (fraction 3). Free PCA-soluble fraction could undergo the dansylation process directly, while the PCA-soluble conjugated fraction had to be processed via acidic hydrolysis, prior to dansylation. The pellet was hydrolyzed by adding 200 μ L of 37% HCl and incubation at 110°C for 18–20 h. Afterwards, HCl was evaporated from the tube by heating at 70°C. The residue was than resuspended in 200 μ L of 0.2 M HClO_4_, which made it ready for the dansylation. To extract PCA-insoluble bound polyamines, the pellet was rinsed two times with 1 mL of 0.2 N PCA to remove any trace of soluble polyamine and then dissolved by vigorous vortexing in 200 μL of 1 N NaOH. The mixture was sonicated for 90 min. The next step, acidic hydrolysis, was performed in the same way as described above. The dansylation was done according to the methods (Flores and Galston, 1982). One hundred μ L aliquots of each fraction were added to 10 μ L of 0.5 M diaminohexan (internal standard), 110 μ L (1 volume) of 1.5 M sodium carbonate (Na_2_CO_3_) and 200 μ L dansyl chloride in acetone (7.5 mg/mL). The mixture was incubated at 60°C for 1 h in the dark. 50 μ L of proline (100 mg/mL) was added to the mixture to saturate excessive dansyl chloride. After further incubation for 30 min at 60°C in the dark, the polyamines were extracted with 250 μ L of toluene and vigorous vortexing for 30 s. The mixture separated into two phases, aquatic and organic. The organic upper phase containing polyamines, was collected and dried in speedvac. The polyamine residue was dissolved in 100 μ L of methanol and assayed immediately or stored at −20°C in the dark. Twenty μl of a sample were injected onto a reverse phase LC-18 column protected by a guard column (Alphabon C18, 10 μm; Supelco, Germany). Samples were eluted from the column with a solvent gradient (v/v) of water: methanol changing from 70% to 100% in 15 min at a flow rate of 1 mL/min. (70–80% methanol for 5 min, 80–100% methanol for 10 min and 100–70% methanol for 5 min). Elution was completed after 25 min. Eluates from the column were detected by an attached fluorescence detector (RF 2000, Dionex). For the dansylated polyamines, an excitation wavelength of 365 nm was used with an emission wavelength of 519 nm.

### Ion chromatography

Free ions (sulfate, nitrate and phosphate) were separated and quantified by the Dionex ICS-2000 Ion Chromatography System (ICS-2000), which performs ion analyses using suppressed conductivity detection. Around 50 mg of the powdered frozen plant material was used for the 1:5 extraction of ions with 0.1 mM HCl. After vigorous vortexing, the samples were centrifuged for 5 min at 14000 rpm at 4°C. The supernatant was collected, centrifuged again and the second supernatant was filtered through the Ultrafree MC 5000 NMWL Filter Unit (Milipore) at 5000 g at 4°C. The samples were stored in −20°C or measured immediately after adjusting the ions concentration range by 1:20 dilution with deionized water. The eluent gradient was increasing over each sample measurement up to 23 mM KOH. For the maximum eluent gradient the suppressor current was 20 mA.

### Chemical element analysis

The chemical element measurements were performed using between 181 and 264 mg of dry plant material digested with 85:15 (v/v) mixture of nitric acid (Primar, Aristar s.g 1.42, 70%) and PCA (Aristar/Primar, 70%) as described previously (Shinmachi et al., 2010), using Inductively Coupled Plasma-Atomic Emission Spectroscopy (ICP-OES, Applied Research Laboratories, Vallaire, Ecublens, Switzerland).

### Metabolome analysis by *metanomics*

Metabolite contents in this study have been determined by *Metanomics* Company (www.metanomics.de), which operates two highly complementary mass-spectrometry technologies, allowing the reliable monitoring of the wide range of chemical classes of metabolites.

### Statistical data evaluation

All statistical analysis was carried out using Excel (Microsoft Office 2003). Significance of differences between means of data sets was determined using the student's *t*-test (heteroscedastic and double-sided). Differences between data sets were regarded as significant when probability of error was below 5% (*P* < 0.05).

### Conflict of interest statement

The authors declare that the research was conducted in the absence of any commercial or financial relationships that could be construed as a potential conflict of interest.
