# Supplementary material for: Transcriptome and metabolome analysis of plant sulfate starvation and resupply provides novel information on transcriptional regulation of metabolism associated with sulfur, nitrogen and phosphorus nutritional responses in Arabidopsis
Source: Front Plant Sci. 2015 Jan 28;5:805. doi: 10.3389/fpls.2014.00805 (PMC4309162; doi:10.3389/fpls.2014.00805)
Supplement: Supplementary file 6 [file Table6.DOCX]

Supplemental Table SVI. Sterile full nutrition and low-sulfate medium composition

|  | Full nutrition (FN) | 150 μM sulfate | Low sulfate (-S) |
| --- | --- | --- | --- |
| Compound | Final [mM] | Final [mM] | Final [mM] |
| KNO_3_ | 2 | 2 | 0.1 |
| NH_4_NO_3_ | 1 | 1 | 0.05 |
| KH_2_PO_4_/K_2_HPO_4_ (pH 5.8) | 3 | 3 | 3 |
| CaCl_2_ | 4 | 4 | 4 |
| MgSO_4_ | 1 | 0.15 | 0 |
| K_2_SO_4_ | 2 | 0 | 0 |
| MgCl_2_ | 0 | 0.85 | 1 |
| KCl | 0 | 4 | 4 |
| MES (pH5.8) | 3 | 3 | 3 |
| Microelements | 1x * | 1x * | 1x * |
| Sucrose | 0.5% | 0.5% | 0.5% |
| Glutamine | 1 | 1 | 1 |

*Microelements: 40 µM Na_2_FeEDTA, 60 µM H_3_BO_3_, 14 µM MnSO_4_, 1 µM ZnSO_4_, 0.6 µM CuSO_4_, 0.4 µM NiCl_2_, 0.3 µM HMoO_4_, 20 nM CoCl_2_
