# Supplementary material for: Transcriptome and metabolome analysis of plant sulfate starvation and resupply provides novel information on transcriptional regulation of metabolism associated with sulfur, nitrogen and phosphorus nutritional responses in Arabidopsis
Source: Front Plant Sci. 2015 Jan 28;5:805. doi: 10.3389/fpls.2014.00805 (PMC4309162; doi:10.3389/fpls.2014.00805)
Supplement: Supplementary file 7 [file Table7.DOCX]

**Supplemental Table SVII. HPLC gradient for thiol analysis**

(A) Elution protocol for thiols; the composition of the current elution solutions is described in %B of A, at the time in min; flow and temperature are constant.

| time [min] | B [%] | flow [ml/min] | temp [°C] |
| --- | --- | --- | --- |
| 0 | 0 | 1 | 25 |
| 2.0 | 0 | 1 | 25 |
| 12.0 | 8 | 1 | 25 |
| 17.0 | 14 | 1 | 25 |
| 19.0 | 100 | 1 | 25 |
| 30.0 | 100 | 1 | 25 |
| 31.5 | 0 | 1 | 25 |
| 35.0 | 0 | 1 | 25 |

(B) Composition of elution solvents for thiols separation on HPLC.

| elution solvent | methanol [%; v/v] | acetic acid [%; v/v] | pH |
| --- | --- | --- | --- |
| A | 10 | 0.25 | 3.9 |
| B | 90 | 0.25 | 3.9 |
