# Supplementary material for: Transcriptome and metabolome analysis of plant sulfate starvation and resupply provides novel information on transcriptional regulation of metabolism associated with sulfur, nitrogen and phosphorus nutritional responses in Arabidopsis
Source: Front Plant Sci. 2015 Jan 28;5:805. doi: 10.3389/fpls.2014.00805 (PMC4309162; doi:10.3389/fpls.2014.00805)
Supplement: Supplementary file 8 [file Table8.DOCX]

**Supplemental Table SVIII. HPLC gradient for amino acid analysis**

(A) Elution protocol, describing the composition of the current elution solution in %B of A at the time in min.

| time [min] | B [%] | flow [ml/min] | temp [°C] |
| --- | --- | --- | --- |
| 0 | 0 | 0.8 | 30 |
| 2.00 | 0 | 0.8 | 30 |
| 16.00 | 13 | 0.8 | 30 |
| 23.25 | 15 | 0.8 | 30 |
| 32.30 | 50 | 0.8 | 30 |
| 43.30 | 60 | 0.8 | 30 |
| 49.30 | 100 | 0.8 | 30 |
| 51.30 | 100 | 0.8 | 30 |
| 58.30 | 0 | 0.8 | 30 |
| 60.00 | 0 | 0.8 | 30 |

(B) Composition of elution solvents for amino acid measurement elution solvent.

| elution solvent | tetra hydrofurane  [%; v/v] | methanol  [%; v/v] |  | acetonitrile  [%; v/v] | sodium phosphate buffer  [mM] | pH |
| --- | --- | --- | --- | --- | --- | --- |
| A | 0.2 | 0 |  | 0 | 8.5 | 6.8 |
| B | 0 | 32.5 |  | 20.5 | 18.5 | 6.8 |
