# Supplementary material for: Transcriptome and metabolome analysis of plant sulfate starvation and resupply provides novel information on transcriptional regulation of metabolism associated with sulfur, nitrogen and phosphorus nutritional responses in Arabidopsis
Source: Front Plant Sci. 2015 Jan 28;5:805. doi: 10.3389/fpls.2014.00805 (PMC4309162; doi:10.3389/fpls.2014.00805)
Supplement: Supplementary file 9 [file Presentation1.PPTX]

## Slide 1
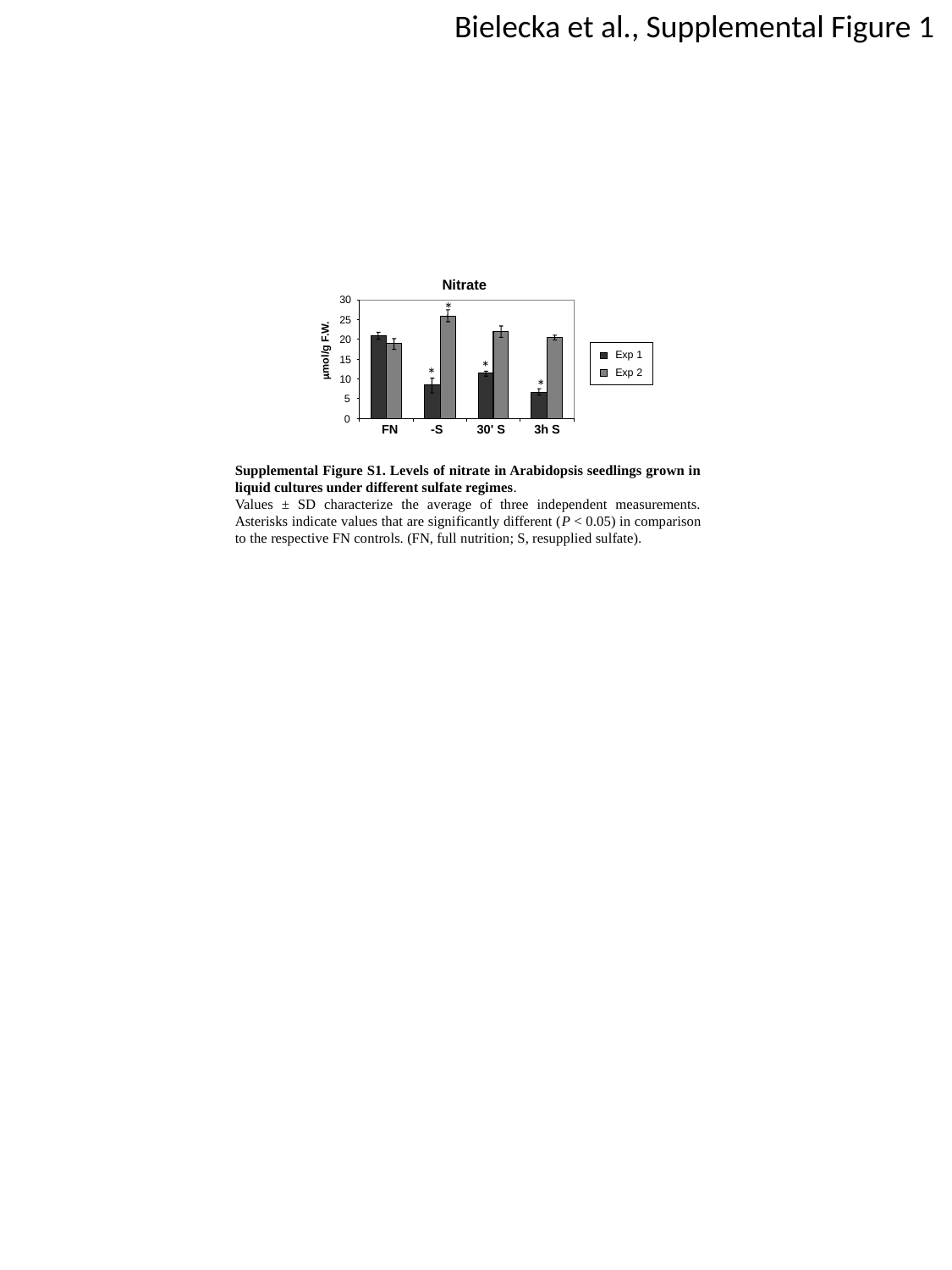

Bielecka et al., Supplemental Figure 1
Nitrate
*
30
25
20
15
10
5
0
*
*
*
Exp 1
Exp 2
mmol/g F.W.
FN
-S
30' S
3h S
Supplemental Figure S1. Levels of nitrate in Arabidopsis seedlings grown in liquid cultures under different sulfate regimes.
Values ± SD characterize the average of three independent measurements. Asterisks indicate values that are significantly different (P < 0.05) in comparison to the respective FN controls. (FN, full nutrition; S, resupplied sulfate).
