# Supplementary material for: Transcriptome and metabolome analysis of plant sulfate starvation and resupply provides novel information on transcriptional regulation of metabolism associated with sulfur, nitrogen and phosphorus nutritional responses in Arabidopsis
Source: Front Plant Sci. 2015 Jan 28;5:805. doi: 10.3389/fpls.2014.00805 (PMC4309162; doi:10.3389/fpls.2014.00805)
Supplement: Supplementary file 10 [file Presentation2.PPTX]

## Slide 1
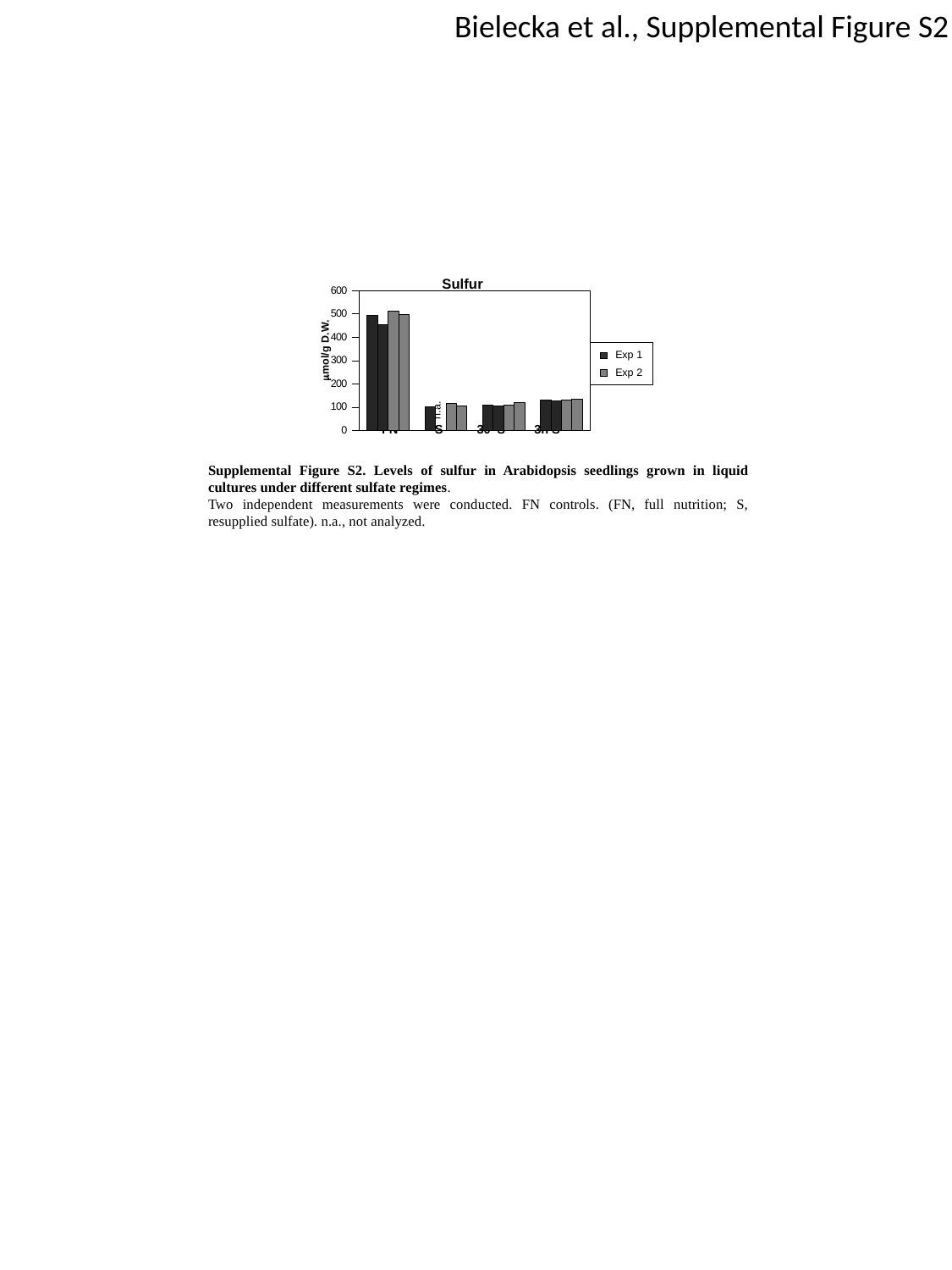

Bielecka et al., Supplemental Figure S2
Sulfur
### Chart
| Category | | | | |
|---|---|---|---|---|
Exp 1
Exp 2
mmol/g D.W.
n.a.
FN
-S
30' S
3h S
Supplemental Figure S2. Levels of sulfur in Arabidopsis seedlings grown in liquid cultures under different sulfate regimes.
Two independent measurements were conducted. FN controls. (FN, full nutrition; S, resupplied sulfate). n.a., not analyzed.
